# Supplementary material for: Causal Relationship between Aging and Anorexia Nervosa: A White-Matter-Microstructure-Mediated Mendelian Randomization Analysis
Source: Biomedicines. 2024 Aug 16;12(8):1874. doi: 10.3390/biomedicines12081874 (PMC11351342; doi:10.3390/biomedicines12081874)
Supplement: Supplementary file 1 [file biomedicines-12-01874-s001.zip › Supplementary Figure/Figure S9/Leaveoneout plot of MR analysis for ubm-b-1478 to AN.pdf]

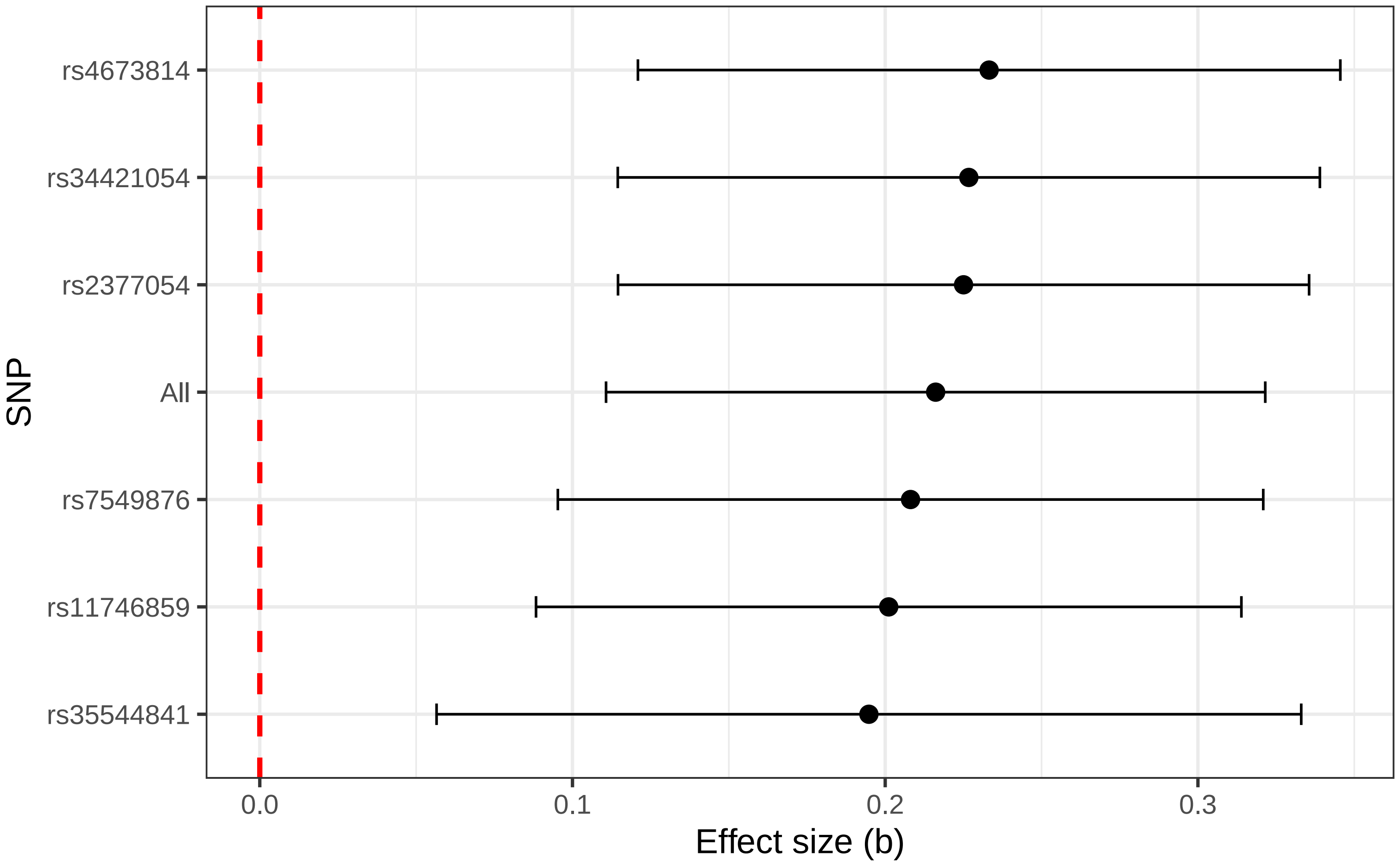

rs4673814
rs34421054
rs2377054
All
rs7549876
rs11746859
rs35544841
SNP
0.0
0.1
0.2
0.3
Effect size (b)
